# Inhibition of Scarb1 on Endothelial Cells Attenuates Pressure Overload-Induced Heart Failure Progression

**DOI:** 10.1016/j.jacbts.2025.05.003

**Published:** 2025-08-06

**Authors:** Toshiomi Katsuki, Dai Kusumoto, Yohei Akiba, Mai Kimura, Jin Komuro, Takahiro Nakamura, Hisayuki Hashimoto, Thukaa Kouka, Kazuhisa Sugai, Yoshinori Katsumata, Masaki Miyasaka, Yutaka Suzuki, Junko Kuramoto, Yoshiaki Kubota, Keiichi Fukuda, Shinsuke Yuasa, Masaki Ieda

**Affiliations:** a Department of Cardiology, Keio University School of Medicine, Shinjuku-ku, Tokyo, Japan; b Department of Cardiology, Saitama City Hospital, Midori-ku, Saitama-shi, Saitama, Japan; c Center for Preventive Medicine, Keio University School of Medicine, Shinjuku-ku, Tokyo, Japan; d Department of Biomedical Informatics and Molecular Biology, The Sakaguchi Laboratory, Keio University School of Medicine, Shinjuku-ku, Tokyo, Japan; e Institute for Integrated Sports Medicine, School of Medicine, Keio University, Shinjuku-ku, Tokyo, Japan; f Department of Computational Biology and Medical Sciences, the University of Tokyo, Kashiwa, Japan; g Department of Pathology, Keio University School of Medicine, Shinjuku-ku, Tokyo, Japan; h Department of Anatomy, Keio University School of Medicine, Shinjuku-ku, Tokyo, Japan; i Department of Cardiovascular Medicine, Faculty of Medicine, Dentistry and Pharmaceutical Sciences, Okayama University, Okayama, Japan

**Keywords:** endothelial cells, fibroblasts, heart failure, SCARB1

## Abstract

Inappropriate endothelial cell (EC) interactions contribute to heart failure; however, their precise mechanisms remain poorly understood. This study investigated EC-fibroblast interactions mediated by Scarb1 using single-cell RNA-sequencing analysis in a mouse heart failure model. ECs exhibited inflammatory and fibrotic gene expression, with Scarb1-mediated fibroblast-EC interactions driving disease progression. EC-specific Scarb1 knockout and systemic SCARB1 inhibition attenuated heart failure progression. In vitro and spatial omics analyses confirmed the role of SCARB1 in ECs and cell-cell interaction during heart failure progression. These findings highlight SCARB1 as a promising therapeutic target for EC-focused interventions.

## Highlights

• scRNA-seq of mouse hearts subjected to pressure overload by TAC revealed that ECs exhibit inflammatory and fibrotic gene expression profiles during heart failure progression.
• Interactome analysis indicated that pathologic interactions between FBs and ECs, mediated by the Scarb1 receptor on ECs, play a pivotal role in heart failure progression.
• Both EC-specific Scarb1 knockout and pharmacologic inhibition of SCARB1 significantly improved cardiac function and reduced fibrosis in pressure overload-induced heart failure models.
• Spatial omics and coculture experiments confirmed that pathologic FB-EC interactions contribute to the pathogenesis of heart failure.

## Visual Abstract

**Figure:**
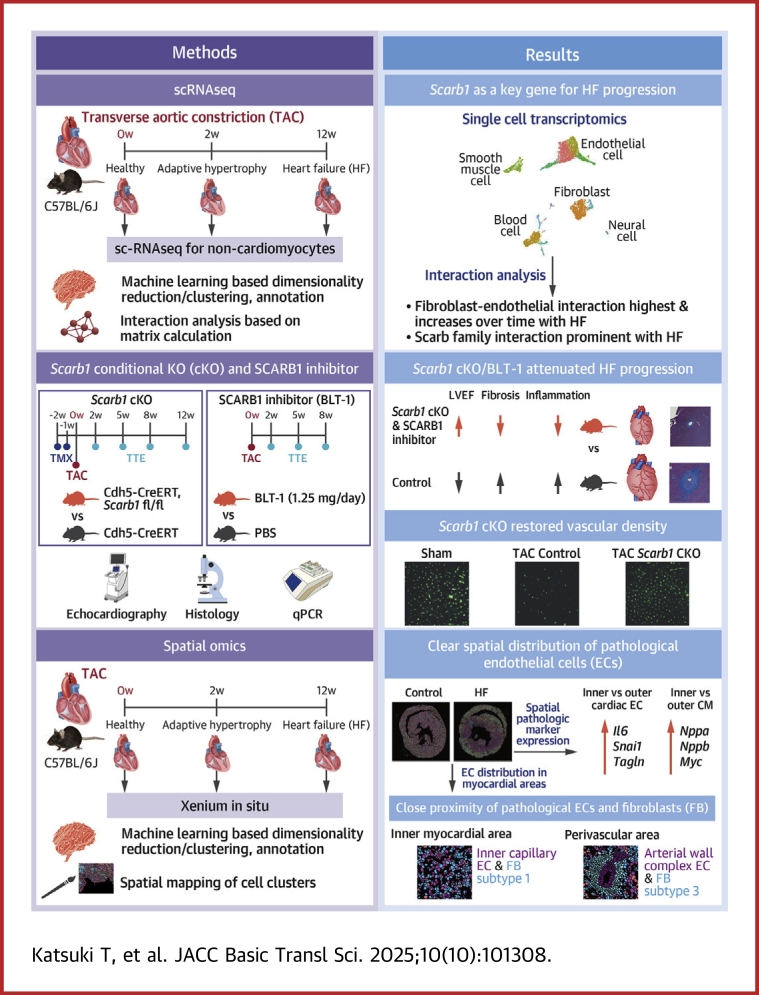

Heart failure significantly contributes to shortened global healthy life expectancy.^1^ Although there are various cell types in the heart, previous studies have focused on cardiomyocytes (CMs), given their crucial roles, including pumping blood in the heart.^2^, ^3^, ^4^, ^5^ Non-CMs, which comprise nearly 70% cells in the heart,^6^ significantly affect heart failure progression.^7^, ^8^, ^9^ Among non-CMs, the endothelial cell (EC) population is the largest.^6^ ECs play important roles in maintaining cardiac homeostasis;^10^, ^11^, ^12^ EC dysfunction contributes to heart failure progression.^13^, ^14^, ^15^ Because EC-specific gene modifications improved heart failure progression,^16^^,^^17^, they are a potent therapeutic target for heart failure. However, the detailed mechanisms of EC dysfunction and its contribution to heart failure progression remain unclear. Furthermore, in heart failure progression, pathologic changes in cells occur at the cellular level and through interactions between multiple cells, including ECs.^18^, ^19^, ^20^, ^21^ Although many previous studies have demonstrated the importance of cell-cell interactions via CMs,^18^^,^^20^^,^^22^ fibroblast (FB)- EC interactions also play a significant role in the heart. FB-EC interactions contribute to the maintenance of heart homeostasis,^23^ and their maladaptive interaction has been reported to be associated with heart disease pathology.^24^^,^^25^ However, the detailed mechanisms by which FB-EC interactions contribute to the progression of heart failure remain poorly understood. Single-cell RNA-sequencing (scRNA-seq) enables a comprehensive analysis of the transcriptional profiles of various cells within tissues at the single-cell level and can reveal cell-cell interactions by calculating ligand-receptor pair expression.^26^, ^27^, ^28^

In this study, we performed scRNA-seq analysis targeting non-CMs through heart failure progression to elucidate the pathologic changes in cells, mainly focusing on ECs and FBs and their pathologic cell-cell interactions. Furthermore, we focused on Scarb1 expressed in ECs based on cell-cell interaction analysis and proposed that Scarb1 could be a novel therapeutic target for heart failure.

## Methods

### Animals

The animal procedures followed the Guide for the Care and Use of Laboratory Animals published by the US National Institutes of Health (Publication no. 85-23, revised 1996), and the study protocol was approved by the Institutional Animal Care and Use Committee of Keio University School of Medicine. *Scarb1* flox mice (C57BL/6J background) were provided by Dr Thierry Huby (Sorbonne Université).^29^ *Cdh5*-CreERT2 mice were purchased from the Center for Animal Resources and Development, Kumamoto University. These mice were originally developed by Dr Kubota (Keio University Hospital).^30^ All mice were housed in climate-controlled (23 °C), specific pathogen-free facilities with a 12-hour light-dark cycle and free access to standard laboratory food (CE2, CLEA Japan Inc) and water at Keio University.

### Transverse aortic constriction

Inhalation anesthesia with 4% isoflurane (Fujifilm) was administered to 8-week-old male C57BL/6J JCL mice (Nihon CREA). After the mice were sedated, 1% isoflurane was administered. A 22-gauge outer cannula (Terumo) was inserted into the trachea. A respirator was connected to the cannula and artificial breathing was established by 0.1 mL × 120 breaths/min. The mice were placed in the supine position and their chest hair was removed using an epilation cream (Kracie). The chest walls were gently washed and sterilized with 70% ethanol (Kaneichi). An approximately 2-cm midline skin incision was made on the chest. The second intercostal space was opened and the thymus was pushed toward the head. Fat and connective tissues were carefully detached from the aortic arch. The arch was ligated using a 10-0 nylon thread (Natsume) with 29-gauge needle (Nipro) spacer. Two knots were made and atrial enlargement was observed. The spacer was removed, and blood flow resumption was confirmed by atrial contraction. The opened intercostal space and skin were sutured using 5-0 silk thread (Natsume). Anesthesia was suspended, and intubation was removed after the recovery of spontaneous breathing. The mice were allowed to recover on a 37 °C heated pad.

### Echocardiography

Inhalation anesthesia was introduced and the hair on the chest wall was removed, similar to the transverse aortic constriction (TAC) procedure. First, a parasternal long-axis view was obtained, and the optimal echo probe (Vevo 2100, FujiFilm VisualSonics) position was adjusted. Next, the probe was rotated just above the papillary muscles and a short-axis view was obtained. An M-mode video was recorded, and wall thickness parameters (interventricular septum thickness; posterior left ventricular wall thickness) and left ventricular chamber diameters (left ventricular end-diastolic dimension and internal dimension in systole) were measured. The left ventricular ejection fraction (LVEF) was estimated by these formulas^31^:

$$\text{EDV}=\left\{7.0\;/\;\left(2.4+\text{LVDd}\right)\times {\text{LVDd}}^{3}\right\}$$

$$\text{ESV}=\left\{7.0\;/\;\left(2.4+\text{LVDs}\right)\times {\text{LVDs}}^{3}\right\}$$

LVEF={100×(EDV-ESV)/EDV}

where EDV is end-diastolic volume; ESV, end-systolic volume; LVDd, LV end-diastolic dimension; and LVDs, LV internal dimension in systole.

Echocardiography was performed at 0, 2, 5, and 8 weeks after TAC in block lipid transport-1 (BLT-1, Sigma-Aldrich)–administered mice, and at 0, 2, 5, 8, and 12 weeks after TAC in *Scarb1* conditional knockout (CKO) mice.

### Preparation of single-cell suspension

Mice were euthanized using the cervical dislocation technique. The rib cage was quickly opened with scissors and 10 mL of ice-cold phosphate-buffered saline (PBS) was intermittently injected into the right ventricle after the left atrium incision. When the lungs turned white, the hearts were extracted and soaked in ice-cold PBS. The atria were removed in cold PBS. Type II collagenase (Worthington) (13.5 g) and DNase I (2 mg, Qiagen) were dissolved in 30 mL of F12Ham medium (Thermo Fisher Scientific). The heart was minced using scissors in an ice-cold Petri dish (Corning) and mixed with a collagenase solution. The solution was poured into an Erlenmeyer flask soaked in a 38 °C warm bath (Taitec) with a magnetic stirrer (AS ONE) for 30 minutes. After a 30-minute incubation, the mixture was repeatedly cannulated using an 18-gauge needle (Nipro) with a 10-mL syringe (Terumo). After cannulation, a 30-minute reaction was performed. Ice-cold PBS (20 mL) was used to stop the reaction. This solution was centrifuged at 450*g* and 4 °C. The supernatant was removed, and 5 mL of 1X RBC Lysis Buffer (Thermo Fisher Scientific) was added on ice with gentle shaking. Ice-cold PBS (45 mL) was added and the mixture was passed through 70- and 40-μm filters (pluriSelect), consecutively. The solution was centrifuged as previously described, and the supernatant was removed. CellCover (3 mL, Anacyte Laboratories) was added on ice for 5 minutes. Ice-cold PBS (47 mL) was added and centrifuged as described previously. The supernatant was removed, and 4 mL of ice-cold PBS was added. In the 15-mL tube (Corning), 3 mL “bottom Percoll,” 4 mL “top Percoll,” and the previous solution of 1 mL was stacked in this order. The bottom Percoll and top Percoll solutions are shown in Table 1.

**Table 1:** Bottom and Top Percoll Solutions

Solution	Percoll stock solution	10 X Ads	ddH_2_O
Bottom Percoll	130 mL	7 mL	63 mL
Top Percoll	90 mL	11 mL	99 mL

ddH_2_O = double-distilled H_2_O.

A Percoll stock solution was prepared by mixing 450 mL of Percoll (Sigma-Aldrich) with 50 mL of 10 X Ads. The ingredients of the 10 X Ads are shown in Table 2.

**Table 2:** Ingredients of the 10 X Ads

5 M NaCl	HEPES	1 M NaH_2_PO_4_	1 M Glucose	3 M KCl	1 M MgSO_4_
116 mL	23.8 g	5 mL	27.5 mL	9 mL	4 mL

HEPES = 2-[4-(2-hydroxyethyl)piperazin-1-yl]ethane sulfonic acid; M = mol/L.

The stacked solution was centrifuged for 25 minutes at 3000*g* (Beckman) and 25 °C. The debris layer and top Percoll were swiftly aspirated. The cell layer and bottom Percoll were moved to a new tube and diluted with ice-cold PBS up to 50 mL and centrifuged for 6 minutes at 450*g* and 4 °C. The supernatant was removed, and the precipitate was washed in the same manner. Finally, 1 mL of CellCover was added before application to the single-cell analysis apparatus.

### scRNA-seq, mapping, and exploratory analysis

This solution was applied to a Chromium Controller (10x Genomics) using a manufacturer-defined protocol.^32^ The RNA solution was then applied to the HiSeq 3000 system (Illumina). Read count data were processed using Cell Ranger (10x Genomics) and analyzable unique molecular identifier count data were retrieved.

We used R 4.2.1 (R Foundation), Seurat 4.1.0,^33^ Monocle 3 (version 1.3.1),^34^ and circlize 0.4.15 packages.^35^ To convert unique molecular identifier count data into analyzable “Seurat object,” the “Read10X” and “CreateSeuratObject” functions in the Seurat package were used. The options for the latter were minimum cell=3 and minimum feature=200. “NormalizeData” function in the Seurat package was used for data normalization. To each data set (TAC: 0 week, 2 weeks, 12 weeks), the “FindVariableFeatures” function in the Seurat package was applied to extract key genes for clustering cells that are called “features.” The options were selection.method=“vst” and nfeatures=2000. To remove the batch effect, the Seurat package employs “anchor” approach that uses metric learning. “SelectIntegrationFeatures” function selected key genes for “anchor” cells that were utilized for connecting nodes of different data sets. “FindIntegrationAnchors” function used these genes to identify anchor cells. Finally, the “IntegrateData” function integrated the 3 different timing data sets into 1. The integrated data sets were scaled, dimensionally reduced, and clustered by “ScaleData,” “RunPCA,” “RunUMAP,” “FindNeighbors,” and “FindClusters” functions. Specifically, the FindNeighbors function constructs a k-nearest neighbor graph (k = 20) and converts it into a shared nearest neighbor graph. The FindClusters function then clusters this graph by optimizing a modularity function using the Louvain algorithm (Github). After the first clustering, crude cell type populations (ECs, smooth muscle cells, FBs, blood cells, and neural cells) were identified by known cell-type specific markers (“Pecam1,” “Cdh5,” “Kdr,” “Myh11,” “Tagln,” “Acta2,” “Pdgfra,” “Col1a1,” “Tcf21,” “Ptprc,” “Itgam,” “Kcna1,” “Kcna2,” and “Sox10”). For further investigation of the subtypes, we divided the combined data set into sub–data sets of each crude cell type and reclustered them using the same settings. Reannotation was performed using the genes shown in the figures. Specifically ECs were annotated by “Fabp4,” “Fabp5,” “Car4,” “Aplnr,” “Dll4,” “Hey1,” “Unc5b,” “Gja4,” “Gja5,” “Nr2f2,” “Vwf,” “Top2a,” “Birc5,” “Lyve1,” and “Prox1.” The Chiefly Monocle 3 package was used to visualize local gene expression transitions and cell trajectories. The initial points of the trajectories were determined based on the cell property. Seurat data objects were converted into “cell_data_set” objects. They were clustered, embedded in graphs, and aligned using the Monocle 3 function as a vignette showed (Github).

### Gene ontology analysis

To identify key genes that discriminate certain populations from others in scRNA-seq analysis, we employed “FindMarkers” function in the Seurat package. The output genes were screened using a threshold of *P* < 0.05 (Bonferroni), and the top 150 genes were cast into the DAVID (Database for Annotation, Visualization, and Integrated Discovery) webapp. The Gene Ontology (GO) (biological process, cellular component, and molecular function) and KEGG (Kyoto Encyclopedia of Genes and Genomes) pathway results were extracted.

Similarly, the output genes of RNA-seq analysis of human cardiac microvascular endothelial cells (HMVECs) were screened by a threshold of *P* < 0.05 (Benjamini and Hochberg). Up-regulated genes (fold-change > 2; 183 genes) or down-regulated genes (fold-change <0.5; 95 genes) were used in the DAVID analysis.

### Interaction analysis

To quantify cell-cell interactions, we conducted matrix calculations. We sliced the known ligand and receptor matrix^36^ from the raw unique molecular identifier count matrix. The expression of each gene was averaged within each sample week (0 week, 2 weeks, and 12 weeks) or cell-count–weighted averaged through subtypes, depending on the subsequent analysis. These expression matrices were scaled by the sum of the matrices themselves and multiplied by 100,000. Given that the ligand column vector was *L* and the receptor row vector was *R*, the adjacent matrix of each cell type (*A*) was calculated as follows: *A* = *L* × *R* (noninterchangeable). These ligand-receptor interactome were visualized using the circlize package as circos plots.

### Scarb1 receptor blocker treatment of TAC mice

TAC was performed as previously described. Mice were randomized into treatment or control groups. Fifty microliters of BLT-1 (Sigma-Aldrich) solution in 1 mL PBS was intraperitoneally injected into the treatment group mice once a day. The BLT-1 solution consisted of 25 mg of BLT-1 and 1 mL of dimethyl sulfoxide (DMSO). Echocardiography was performed during preoperation and at postoperative weeks 2, 5, and 8.

### Quantification of HDL in vivo

To test the effect of BLT-1 on the plasma concentration of high-density lipoprotein (HDL), we collected blood samples from the right atrium of sacrificed mice. SRL Inc quantified the HDL concentrations in these samples.

### Conditional knockout of *Scarb1* in ECs

*Cdh5*-CreERT2/*Scarb1*^flox/flox^ and *Cdh5*-CreERT2/ *Scarb1*^+/+^ mice were generated. Tamoxifen oil was intraperitoneally injected (45 mg/kg body weight) into these mice 2 and 1 weeks before the TAC operation. Tamoxifen oil was prepared by diluting 50 mg tamoxifen (Sigma-Aldrich) in 1 mL ethanol (Wako) and 9 mL sunflower oil (Sigma-Aldrich). TAC was performed as previously described. Echocardiography was performed during preoperation and at postoperative weeks 2, 5, 8, and 12.

### Fibrosis quantification

In the azan-stained specimen, the perivascular fibrosis ratio was calculated as follows: Fibrosis ratio = A_F_/abπ (no unit), given A_F_ was the area of fibrosis, a/b was the long-/short-axis lengths of the target vessel, and π was the circular constant. Meanwhile, the interstitial fibrosis ratio was calculated as follows: Fibrosis ratio = A_F_/A_FoV_ (no unit), given A_F_ was the area of fibrosis, and A_FoV_ was the area of the field of view.

### Reverse transcription polymerase chain reaction

Total RNA was dissolved in TRIZOL (Thermo Fisher Scientific), separated using chloroform (Wako), precipitated with isopropyl alcohol (Wako), and purified using 70% ethanol (Wako). ReverTra Ace qPCR RT Master Mix with gDNA Remover (Toyobo) was used for reverse transcription. The primers used are shown in Table 3.

**Table 3:** Primers Used for Reverse Transcription

	Forward	Reverse
Human		
Gapdh	GGAGCGAGATCCCTCCAAAAT	GGCTGTTGTCATACTTCTCATGG
Acta2	TGGCATTGCCGACCGAAT	GTGGACAGAGAGGCCAGGAT
S100a4	CTCTACAACCCTCTCTCCTCG	CTCATCAGCTTCTGGAAAGCA
Mouse		
*Gapdh*	AGCCTCGTCCCGTAGACAAA	CCTTGACTGTGCCGTTGAAT
*Postn*	CCTGCCCTTATATGCTCTGCT	AAACATGGTCAATAGGCATCACT
*Col1a1*	GCTCCTCTTAGGGGCCACT	CCACGTCTCACCATTGGGG
*Il1b*	ATCTTTTGGGGTCCGTCAACT	GCAACTGTTCCTGAACTCAACT
*Il6*	TAGTCCTTCCTACCCCAATTTCC	TTGGTCCTTAGCCACTCCTTC
*Tgfb*	CTCCCGTGGCTTCTAGTGC	GCCTTAGTTTGGACAGGATCTG
*Timp1*	GCAACTCGGACCTGGTCATAA	CGGCCCGTGATGAGAAACT
*Nppa*	GGGGGCATGACCTCATCTT	GCTTCCAGGCCATATTGGAG
*Nppb*	GAGGTCACTCCTATCCTCTGG	GCCATTTCCTCCGACTTTTCTC
*Scarb1*	TTTGGAGTGGTAGTAAAAAGGGC	TGACATCAGGGACTCAGAGTAG

### Coculture of ECs and FBs

HMVECs (HMVEC Cardiac MV Endo, batch number 0000550176, Lonza) and rat heart FBs separated from 3- to 7-day-old Jcl:Wistar rats (CLEA Japan Inc) were prepared. In the mixed coculture group, they were seeded together in each well of a 6-well plate (Corning) by 1 × 10^5^ cells/2.5 mL and 0.5 × 10^5^ cells/2.5 mL final concentrations, respectively. In the monoculture group, HMVECs were seeded at a final density of 2 × 10^5^ cells/2.5 mL. In each treatment well, a final concentration of 5 μmol/L BLT-1 (Sigma-Aldrich) was added. DMSO was added to each control well to ensure that the final DMSO concentration was the same as that in the treatment group. The culture medium used was EGM-2 MV Microvascular Endothelial Cell Growth Medium-2 BulletKit (Lonza). The cells were cultured for 24 hours, and total RNA was collected as described in the reverse transcription polymerase chain reaction protocol.

Separated coculture experiments were also conducted. HMVECs were seeded in 6-well plates as with mixed coculture, and Falcon Permeable Support for 6-well plate with 1.0-μm Transparent PET Membrane (Corning) were inserted into the wells. Rat FBs were seeded on the membrane apart from HMVECs in the coculture group. Only HMVECs were seeded in the monoculture group.

### RNA-seq of BLT-1–treated HMVECS

HMVECs were seeded in a 6-well plate at a density of 1 × 10^5^ cells/2.5 mL concentrations. The medium was the same as that used for the coculture. There were 6 cultured and control treatments. After 48 hours, 1 μmol/L final concentration BLT-1 (Sigma-Aldrich) was added to treatment wells and DMSO was added so that the final DMSO concentration was the same as the treatment group. Total RNA was collected as previously described, and samples were combined into 3 vs 3 for RNA-seq. RNA was purified using the ReliaPrep RNA Cell Miniprep System (Promega). The sequencing and mapping were performed by Macrogen. The differentially expressed genes between the 2 groups (*P* < 0.05) were subjected to gene set enrichment analysis using the Broad Institute website.

### Visualization of HDL dynamics in vivo

To delineate the dynamics of HDL, 30 μg/body 1,1'-dioctadecyl- 3,3,3',3'-tetramethylindocarbocyanine perchlorate labelled HDL (Dil-HDL, KALEN Biomedical) was used. Dil-HDL was retro-orbitally injected into the target mice 6 hours before sacrifice. Mice were sedated with 1% isoflurane through this procedure.

### Immunohistochemistry

The sample mice were euthanized, and the PBS-perfused hearts and livers were harvested as previously described. Cryosections of these organs were obtained using Tissue-Tek O.C.T. compound (Sakura Finetek Japan). The specimens were washed under running tap water and PBS. Antigen retrieval was performed by soaking the samples in citrate buffer (pH 6.0) and boiling them twice in a microwave. Citrate buffer was prepared with 2.95 g trisodium citrate dihydrate (Fujifilm) and 1 L ultrapure water and titrated with 1 mol/L HCl solution (Fujifilm). Finally, Tween 20 (0.5 mL) was added (Sigma-Aldrich).

Targeting endothelial CD31 and SCARB1, Mouse/Rat CD31/PECAM-1 antibody (R&D Systems), and SR-BI Antibody–BSA free (Novus Biologicals) were utilized. The primary antibodies were dissolved in Blocking One-P (Nacalai Tesque) to obtain a final concentration of 1:100 vol/vol. Specimens were incubated at 4 °C overnight. The specimens were then washed thrice with PBS.

When SCARB1 was immunostained, signal amplification was performed using TSA PLUS CYANINE 5.5 (Perkin Elmer), according to the manufacturer’s protocol. Briefly, the internal peroxidase activity was quenched by incubation with hydrogen peroxide (Wako) for 30 minutes at 25 °C before incubation with the primary antibody. Anti-rabbit HRP (Global Life Sciences Technologies Japan) incubation was performed for 30 minutes at 25 °C after the primary antibody incubation. The specimens were washed thrice in PBS and incubated in TSA PLUS working solution for 10 minutes at 25 °C.

Alexa Fluor 488 donkey anti-goat IgG (Life Technologies) and Alexa Fluor 594 chicken anti-rabbit IgG (Life Technologies) were used as secondary antibodies for CD31 and SCARB1, respectively. The secondary antibodies were dissolved in Blocking One P to obtain a final concentration of 1:1,000 vol/vol. The cells were then incubated for 1 hour at 25 °C.

The specimens were washed in PBS 3 times and incubated in 1:2,000 vol/vol DAPI Solution (BD Bioscience) in PBS solution for 5 minutes.

### Xenium in situ

Xenium in situ was conducted according to the protocol described in a previous study.^37^ In summary, 5-μm formalin-fixed paraffin-embedded tissue sections of mouse hearts were mounted on Xenium slides (10x Genomics). Deparaffinization and permeabilization were carried out adhering to manufacturer's instructions. Probes targeting for 300 selected genes were designed and were hybridized at 50 °C overnight. Subsequently, posthybridization wash (37 °C for 30 minutes), ligation (37 °C for 2 hours), and amplification (30 °C for 2 hours) were performed. Autofluorescence quenching and nuclei staining were performed under the dark conditions. The prepared slide underwent fluorescent probe hybridization and imaging by the Xenium Analyzer (on-board analysis: version 1.1.0.2, software: version 1.1.2.4, 10x Genomics). Following these procedures, hematoxylin and eosin staining was performed to the Xenium slides. The resulting images and expression profiles were analyzed by R 4.2.2 and Seurat 5.0.1 package. Left atrium, endocardium, and epicardium cells were excluded in the downstream analysis after visualization.

### THBS1 administration to HMVECs

HMVEC were seeded in a 6-well plate at a density of 2 × 10^5^ cells/2.5 mL concentrations. The medium was the same as that used for the coculture. After 12 hours, the cells were subjected to serum starvation for 6 hours. Subsequently, THBS1 (Sigma-Aldrich) was administered at a final concentration of 2 μmol/L to the THBS1 (+) BLT1 (−) and THBS1 (+) BLT1 (+) groups. Additionally, BLT-1 (Sigma-Aldrich) was added at a final concentration of 1 μmol/L to the THBS1 (+) BLT1 (+) group. DMSO was supplemented to ensure a uniform final concentration across all treatments. Each group comprised 3 biological replicates. After 24 hours, RNA was extracted as previously described, and quantitative polymerase chain reaction (qPCR) was performed.

### Statistical analysis

Data are presented as the mean ± SEM unless otherwise specified. Data normality was assessed using the Shapiro-Wilk test (shapiro.test() function in R 4.2.1). If normality was confirmed (*P* > 0.05), differences between the 2 groups were evaluated by an unpaired, 2-tailed Welch’s *t*-test (t.test() function in R 4.2.1); otherwise (*P* < 0.05 or *P* = 0.05), the nonparametric Wilcoxon rank-sum test was applied (wilcox.test() function in R 4.2.1). The Wilcoxon rank-sum test was always utilized for differential gene expression analysis using the Seurat package (Seurat 4.1.0). The Kaplan-Meier method was used to plot survival curves. *P* < 0.05 was considered statistically significant: ∗*P* < 0.05, ∗∗*P* < 0.01, and ∗∗∗*P* < 0.001.

### Data Availability Statement

The raw and processed scRNA-seq and RNA-seq data supporting the findings of this study have been deposited in the Gene Expression Omnibus under accession numbers GSE293319 (scRNA-seq) and GSE293318 (RNA-seq). All data will become publicly available upon publication.

## Results

### Overview of non-CMs in the heart

**Figure 1:**
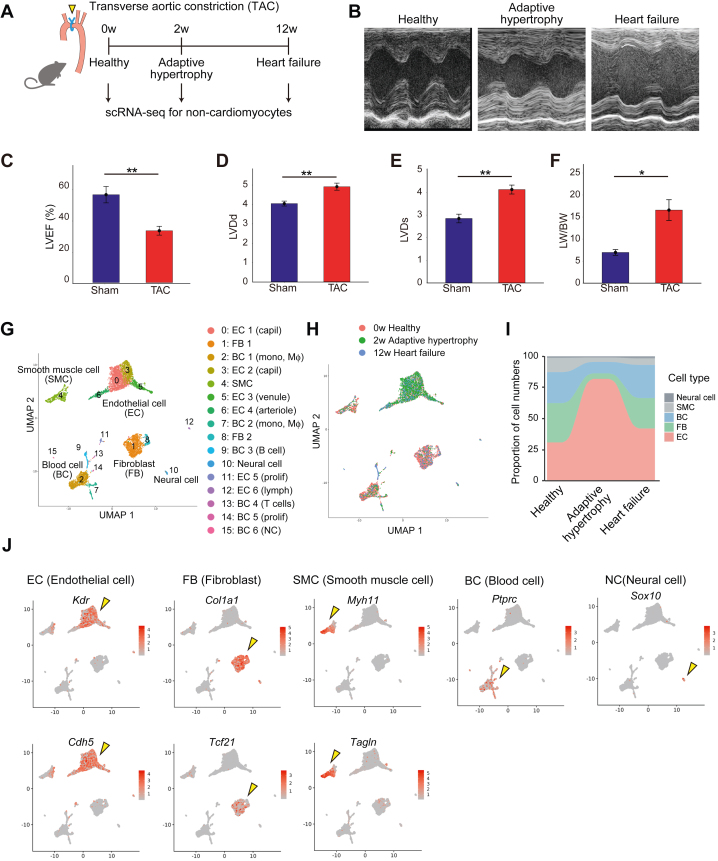
Overview of Non-CMs via Heart Failure Progression Analyzed by scRNA-seq (A) Experimental protocol for single-cell RNA-sequencing (scRNA-seq) analysis. Healthy, adaptively hypertrophic, and failed murine hearts were subjected to scRNA-seq. Two weeks (2w) and 12 weeks after the TAC procedure, adaptive hypertrophy and heart failure phases occurred, respectively. (B) Representative M-mode echocardiograms of sacrificed mice. The healthy (left), adaptive hypertrophy (middle), and heart failure (right) phases are displayed. (C) Left ventricular ejection fraction (LVEF), (D) left ventricular internal diameter at end-diastole (LVDd), (E) left ventricular internal diameter at end-systole (LVDs), and (F) lung weight divided by body weight (LW/BW) parameters of representative sham and transverse aortic constriction (TAC) mice. (G,H) Visualization of single-cell transcriptomes from combined data sets of healthy, adaptive hypertrophic, and failed murine heart samples in a uniform manifold approximation and projection (UMAP) dimensional reduction manner (UMAP plot). (I) Proportional changes in crude cell types during heart failure progression. (J) Visualization of cell type–specific unique molecular identifier counts for endothelial cells (ECs), fibroblasts (FBs), smooth muscle cells (SMCs), blood cells (BCs), and neural cells (NCs). Richer red indicated more abundant expression. Yellow arrowheads indicate the most specific subgroup. ∗*P* < 0.05, and ∗∗*P* < 0.01. CM = cardiomyocyte.

To investigate the pathologic changes in non-CMs during heart failure development, we created TAC mouse models.^38^^,^^39^ We extracted non-CM from hearts in the normal phase (TAC 0 weeks), adaptive hypertrophy phase (TAC 2 weeks), and heart failure phase (TAC 12 weeks) in TAC model mice (Supplemental Figures 1A and 1B) and conducted droplet-based scRNA-seq analysis^32^ (Figures 1A and 1B, Supplemental Figures 1C and 1D). Ultrasound images and lung weight data demonstrated that heart failure was successfully induced by TAC-induced pressure overload (Figures 1C to 1F, Supplemental Figures 1E and 1F). We integrated time series data from the 3 phases of heart failure development using gene anchors^40^ and identified cell types based on known gene expression (Figure 1G). The machine learning–based classification algorithm successfully classified the same cell types in different heart failure stages into the same clusters (Figure 1H). ECs, FBs, blood cells, smooth muscle cells, and neural cells were identified in descending order of cell number, and CMs were not included (Figure 1J, Supplemental Figure 1G). The number of ECs increased with heart failure progression, especially in the hypertrophic phase (Figure 1I, Supplemental Figures 1H and 1I), indicating that the disruption of homeostasis by ECs plays a pivotal role in heart failure progression. We reclustered the major populations into subpopulations to analyze detailed pathologic changes in each cellular type during heart failure development.

### Pathological changes in ECs and FBs through heart failure progression

**Figure 2:**
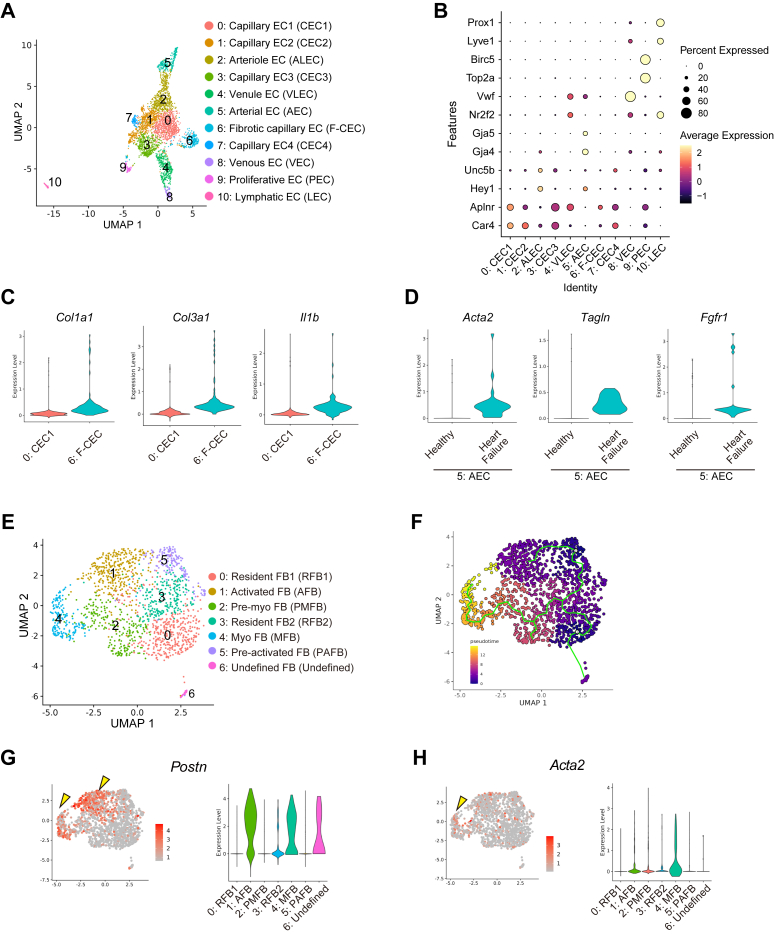
scRNA-seq Revealed Distinct Subgroups and Gene Expression Profiles in ECs and FBs Through Heart Failure Development (A) UMAP plot for reclustered ECs in combined data sets of healthy, adaptively hypertrophic, and failed hearts. (B) Visualization of EC subgroups and their specific marker genes. Larger dots indicate more cells in the subgroups expressing the marker genes. (C) Visualization of the gene expression difference between EC subgroups 0 and 6 by violin plots. (D) Visualization of the gene expression difference in EC subgroup 5 cells in healthy and heart failure phases using violin plots. (E) UMAP plot for reclustered fibroblasts (RFBs) in combined data sets of healthy, adaptively hypertrophic, and failed hearts. (F) Pseudotime overlayed UMAP plot of fibroblasts. Pseudotime was calculated along gene expression similarity of cells. Green lines represent the cell trajectories. Trajectories were aligned using similarity measures, indicating the virtual differentiation of the cells. Brighter colors indicate more differentiated cells. Visualization of key fibroblast gene expression markers for (G) activated fibroblasts (AFBs) and (H) myofibroblasts (MFBs). Yellow arrowheads indicate the characteristic gene expression patterns of activated cells and myofibroblasts. The distributions of gene expression levels in the subgroups were displayed using violin plots. AEC = arterial endothelial cell; ALEC = arteriole endothelial cell; CEC = capillary endothelial cell; F-CEC = fibrotic capillary endothelial cell; LEC = lymphatic endothelial cell; PAFB = pre-activated fibroblast; PEC = proliferative endothelial cell; PMFB = pre-myo fibroblast; VLEC = venule endothelial cell; other abbreviations as in Figure 1.

We first analyzed the changes in ECs, the largest cell population in the heart, during heart failure progression. We reclustered the ECs and classified them into 11 cell populations (Figures 2A and 2B, Supplemental Figures 2A and 2B). By analyzing the clusters that increased in number during heart failure progression, we found that cluster 6 increased during the period of adaptive hypertrophy, whereas cluster 5 increased during the heart failure phase (Supplemental Figure 2C). Cluster 6 was considered as a type of capillary EC based on the gene expression^41^^,^^42^ (Figure 2B). Violin plot analysis revealed increased expression of *Col1a1, Col3a1*, and *Il1b* in cluster 6 (Figure 2C). GO term analysis also showed that the expression of genes related to the extracellular matrix was elevated in cluster 6 (Supplemental Figure 3A). Cluster 5, which increased in number during the heart failure phase, exhibited arterial EC characteristics. When clusters were reanalyzed by heart failure developmental phase, we found that arterial ECs could be divided into 2 groups: healthy and heart failure phases (Supplemental Figure 3B). Stress fiber-related genes, such as *Acta2* and *Tagln* were up-regulated in arterial EC during the heart failure phase (Figure 2D). *Fgfr1*, which plays an important role in organ fibrosis, is also up-regulated in arterial ECs during heart failure (Figure 2D). GO analysis also showed that gene expression related to the actin cytoskeleton was enriched (Supplemental Figure 3C).

Next, we analyzed pathological changes in FB population. FBs were reclustered into 7 subclusters (Figure 2E, Supplemental Figures 4A and 4B). Cluster 0 was abundant in the healthy heart, namely, resident FBs. The pseudotime analysis^43^ of the FB clusters showed that the stem trajectory diverged into cluster 5,1 direction and cluster 2,4 direction with heart failure progression (Figure 2F). Regarding cell numbers, cluster 1 increased during adaptive hypertrophy, whereas cluster 4 increased during heart failure (Supplemental Figure 4C). Extracellular matrix production including *Postn* in FBs increased with heart failure progression and is a key feature of pathologic FB changes in heart failure.^44^^,^^45^ Our analysis revealed that clusters 1 and 4 were *Postn*-positive (Figure 2G), suggesting that these subgroups are pathologic FBs. Moreover, FBs in cluster 4 expressed stress fibers such as *Acta2* (Figure 2H), a unique gene in myofibroblasts.^46^, ^47^, ^48^ According to GO term analysis, genes related to the extracellular matrix and collagen were enriched in activated FBs (Supplemental Figure 5A and 5B).

### EC-FB interaction through heart failure progression

**Figure 3:**
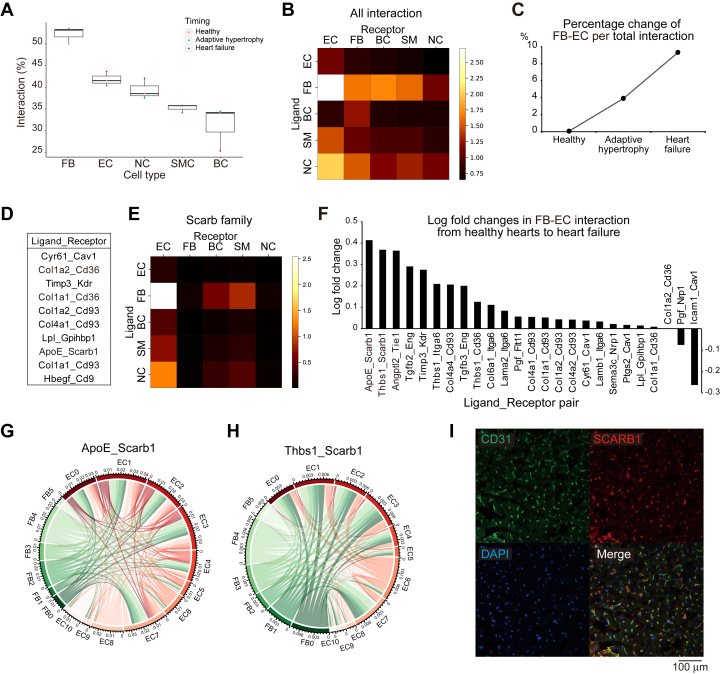
Interaction Analysis of scRNA-seq Data Identified *Scarb1* as a Potential Key Pathological Gene During Heart Failure Progression (A) Boxplot showing the proportion of interactions for each cell type relative to the total interactions. Variances in healthy, adaptive hypertrophy, and heart failure are shown in the boxplots. (B) Heatmap of the total interactions between each cell type. The rows and columns represent ligand-expressing and receptor-expressing cell types, respectively. (C) Percentage change in the total FB-EC interaction during heart failure progression compared to healthy heart. FB-EC means FBs as ligand side and ECs as receptor side. (D) Top-10 strongest interaction pairs between ECs and FBs. We standardized the abbreviations to explicitly display ligand-receptor order, placing the ligand first and the receptor last. (E) Heatmap indicating the total amount of scavenger receptor family interactions between each cell type. (F) Log-fold-changes in FB-EC interaction from the healthy to heart failure phase. In the description of the x-axis, the ligand-receptor order is indicated. The outermost layer bandwidth represents the total amount of interactions provided and received. The green and red bands indicate FBs and ECs, respectively. Arcs between bands represent interactions between cell types. The color and width of the arc correspond to the ligand-expressing cell types and number of interactions, respectively. (G,H) Circos plots of *Scarb1*-specific interaction pairs based on the interactome. (G) ApoE-Scarb1 and (H) Thbs1-Scarb1 interactions. (I) Representative immunohistochemistry images of CD31 (green) and SCARB1(red) in the healthy mouse hearts. Nuclei stained with DAPI (blue). Abbreviations as in Figure 1.

Next, we performed cell-cell interaction analysis using ligand-receptor pairs.^36^ Initially, the absolute number of interactions for each cell type was calculated. We found that the interactions were most active in the following order: FBs, ECs, neural cells, smooth muscle cells, and blood cells (Figure 3A). Next, we analyzed which interactions were most active between cell types and revealed that the interaction between ECs as receptors and FBs as ligands (FB-EC interaction) was the most prominent (Figure 3B). Furthermore, the total number of FB-EC interactions increased with heart failure progression (Figure 3C). Among the receptors in ECs, genes associated with the Scarb family, including *Scarb1* and *Scarb3* (*CD36*), were frequently detected (Figure 3D). When the total number of interactions in the Scarb family was calculated, FBs were found to be the main source of ligands, similar to the total number of interactions (Figure 3E). Finally, we calculated the FB-EC interactions, which increased with heart failure progression, and clarified that the interaction through the SCARB1 receptor^49^ was a top-2 interaction (Figures 3F to 3H). These results suggested that FB-EC interactions via SCARB1 may have an impact on pathologic changes in heart failure development. We also confirmed the focal expression of *Scarb1* in capillary ECs in the heart by immunofluorescence (Figure 3I).

### EC selective knockout of *Scarb1* mitigated heart failure progression

**Figure 4:**
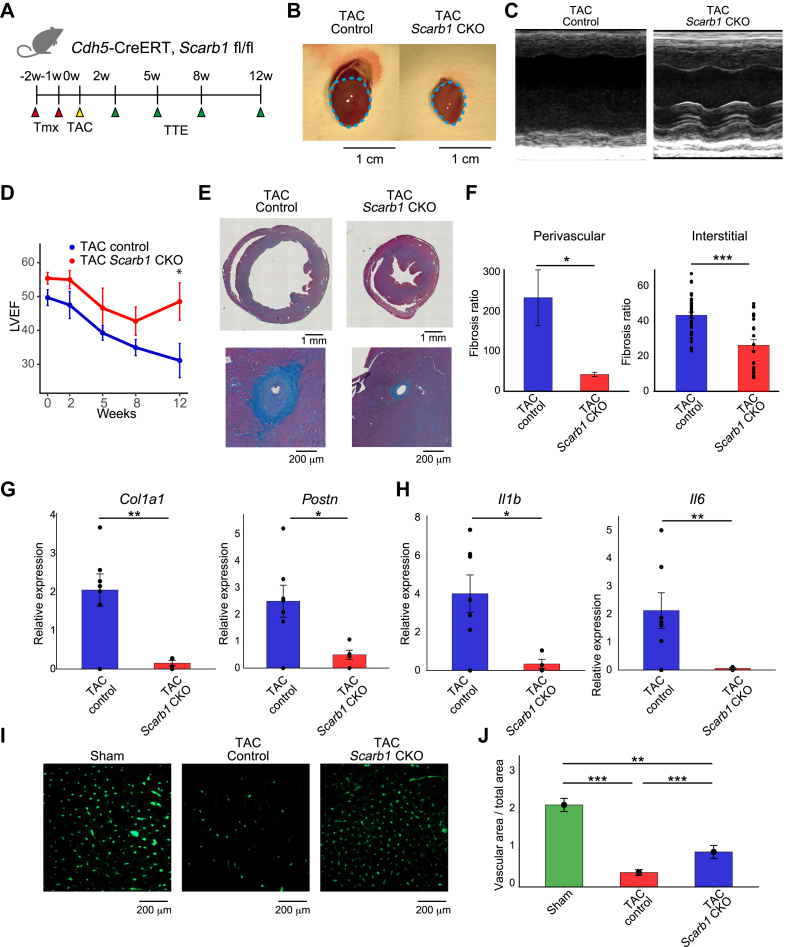
EC-specific Scarb1 KO Ameliorated Heart Failure Condition in Pressure-Overloaded Heart (A) Experimental scheme for EC-specific *Scarb1* conditional knockout (CKO) in TAC mice. Before surgery, at weeks 2 and 1, Tmx was intraperitoneally injected into (*Cdh5*-CreERT, *Scarb1* fl/fl) mice, and (*Cdh5*-CreERT, *Scarb1* +/+) mice. Echocardiography was performed before surgery and at 2, 5, 8, and 12 weeks after surgery. (B) Representative photographs of sacrificed murine hearts. Control (left) and *Scarb1* CKO groups (right). (C) Representative M-mode echocardiography images of control (left) and *Scarb1* CKO mice (right). (D) LVEF changes after TAC surgery. Welch’s *t*-test was performed for the data at the initial week, and Wilcoxon rank-sum test was used for the data at the last week. (E) Representative microscopic images of azan-stained sacrificed hearts. (F) Quantification of the perivascular and interstitial fibrosis ratio in the control and *Scarb1* CKO groups (n = 9 vs 6). Perivascular and interstitial fibrosis ratio was tested using Wilcoxon rank-sum test. Result of quantitative polymerase chain reaction analysis for (G) fibrotic or (H) inflammatory genes in the TAC control and TAC *Scarb1* CKO groups (n = 9 vs 6). Wilcoxon rank-sum test was used to test these data. (I) CD31 immunostaining images of the sham, TAC control, and TAC *Scarb1* CKO groups. (J) Vascular area density of the Sham, TAC control, and TAC *Scarb1* CKO groups. Wilcoxon rank-sum test was used to test between sham/TAC control and Sham/TAC *Scarb1* CKO groups. ∗∗*P* < 0.01, and ∗∗∗*P* < 0.001. Tmx = tamoxifen; TTE = transthoracic echocardiography; other abbreviations as in Figure 1.

scRNA-seq analysis of murine heart failure model derived the hypothesis that FB-EC interaction mediated by SCARB1 receptor could be a key factor in the progression of heart failure. To examine whether *Scarb1* is actually a critical pathologic gene in mouse heart failure models, we generated EC-specific *Scarb1* knockout mice (*Scarb1* CKO) by crossing *Cdh5*-CreERT2 mice expressing EC-specific Cre induced by tamoxifen with *Scarb1*-flox mice (Figure 4A). qPCR analysis demonstrated a significant reduction in *Scarb1* expression in the heart following tamoxifen-induced endothelial-specific knockout, with minor changes observed in liver expression (Supplemental Figure 6A). Heart failure was induced by performing TAC after tamoxifen administration in *Scarb1* CKO and control mice (Figure 4B, Supplemental Figures 6B and 6C). Echocardiography revealed that EC-specific *Scarb1* KO significantly improved LVEF dysfunction compared to that in control TAC mice (Figures 4C and 4D), restoring LVEF to pre-TAC levels and suggesting that Scarb1 in ECs plays a critical role in the progression of TAC-induced heart failure (Supplemental Figure 6D). Furthermore, to investigate whether *Scarb1* CKO reduced cardiac fibrosis, we conducted azan staining of heart tissue. It revealed that both perivascular and interstitial fibrosis was significantly inhibited by *Scarb1* CKO (Figures 4E and 4F). qPCR showed that the fibrotic markers *Col1a1* and *Postn* and the inflammatory molecules *Il1b* and *Il6* were significantly decreased by *Scarb1* CKO (Figures 4G and 4H). Cardiac vasculature is important for maintaining cardiac homeostasis, and it decreases through the progression of decompensated heart failure.^4^^,^^50^^,^^51^ Vascular density decreased following by TAC-induced heart failure, which is alleviated by *Scarb1* CKO (Figures 4I and 4J, Supplemental Figure 6E). Heart weight–body weight ratio was unchanged by EC-specific Scarb1 KO (Supplemental Figure 6F). Similarly, survival rate was unchanged (Supplemental Figure 6G).

### SCARB1 receptor blockade attenuated heart failure progression

**Figure 5:**
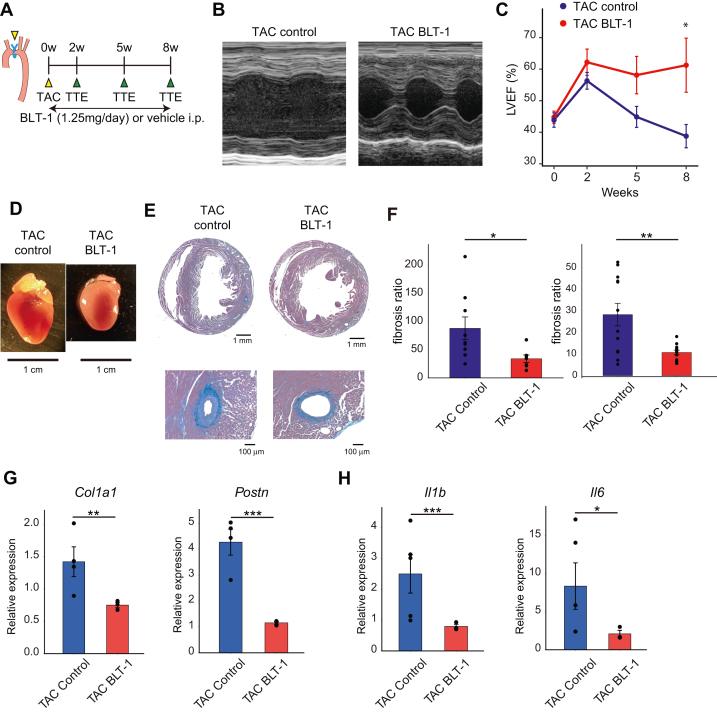
SCARB1 Receptor Inhibitor Attenuated Pressure Overload-induced Heart Failure Progression (A) Experimental schematic of block lipid transport-1 (BLT-1) treatment with TAC. Mice subjected to TAC were randomized into BLT-1 treatment and control groups. From the surgery day, BLT-1 1.25 mg/body/d or phosphate-buffered saline was intraperitoneally (i.p.) injected into the treatment and control groups, respectively. Echocardiography was performed before surgery and at 2, 5, and 8 weeks after surgery. (B) Representative M-mode echocardiography images of control mice (left) and BLT-1 treatment mice (right). (C) LVEF changes after TAC surgery in control mice and BLT-1 treatment mice. (D) Representative photos of sacrificed murine hearts. Control (left) and BLT-1 treatment groups (right). (E) Representative microscopic images of azan-stained sacrificed hearts. (F) Results of the perivascular and interstitial fibrosis ratio quantification in the control and BLT-1 treatment groups (n = 3 vs 3). Result of quantitative polymerase chain reaction analysis for (G) fibrotic or (H) inflammatory genes in the TAC control and TAC BLT-1 groups (n = 4 vs 5). *Col1a1* and *Il1b* were tested using Wilcoxon rank-sum test. *Gapdh* was used as the internal control. Each point represents a single measurement. ∗*P* < 0.05, ∗∗*P* < 0.01, and ∗∗∗*P* < 0.001. Abbreviations as in Figures 1 and 4.

To verify whether SCARB1 receptors in ECs could be the therapeutic target for heart failure, we administered the SCARB1-specific inhibitor, BLT-1^52^ daily to TAC-induced heart failure mice (Figure 5A). Echocardiography revealed that the impairment of cardiac function induced by TAC was rescued in BLT-1 administration (Figures 5B to 5D, Supplemental Figures 7A and 7B). Additionally, both perivascular and interstitial fibrosis was also suppressed in BLT-1–treated mice (Figures 5E and 5F). qPCR showed that the fibrotic markers *Col1a1*, *Postn*, *Tgfb,* and *Timp1*, which were increased by TAC, were significantly decreased after BLT-1 treatment (Figure 5G, Supplemental Figure 7C). Inflammatory molecules, such as *Il1b* and *Il6* were also down-regulated following BLT-1 administration (Figure 5H). Heart weight–body weight ratio increased, indicating transition to decompensated heart failure might be prevented by BLT-1 (Supplemental Figure 7D). Survival rate was unchanged by BLT-1 administration (Supplemental Figure 7E).

Next, we investigated HDL involvement in the observed phenomena, because SCARB1 is a specific HDL uptake receptor.^53^ We used Dil-HDL to visualize HDL uptake into tissues (Supplemental Figures 8A and 8B). In the liver, there was no significant difference in HDL uptake between the BLT-1 administration and control groups (Supplemental Figure 8C), suggesting that HDL uptake was not affected by the BLT-1 dose used in this study. Additionally, we confirmed that HDL uptake did not occur in the heart regardless of BLT-1 administration (Supplemental Figure 8D). Therefore, the cardioprotective effect of BLT-1 may be mediated by direct signaling through SCARB1, rather than an action mediated by HDL.

### Pathological changes in ECs were triggered by direct communication with FBs

**Figure 6:**
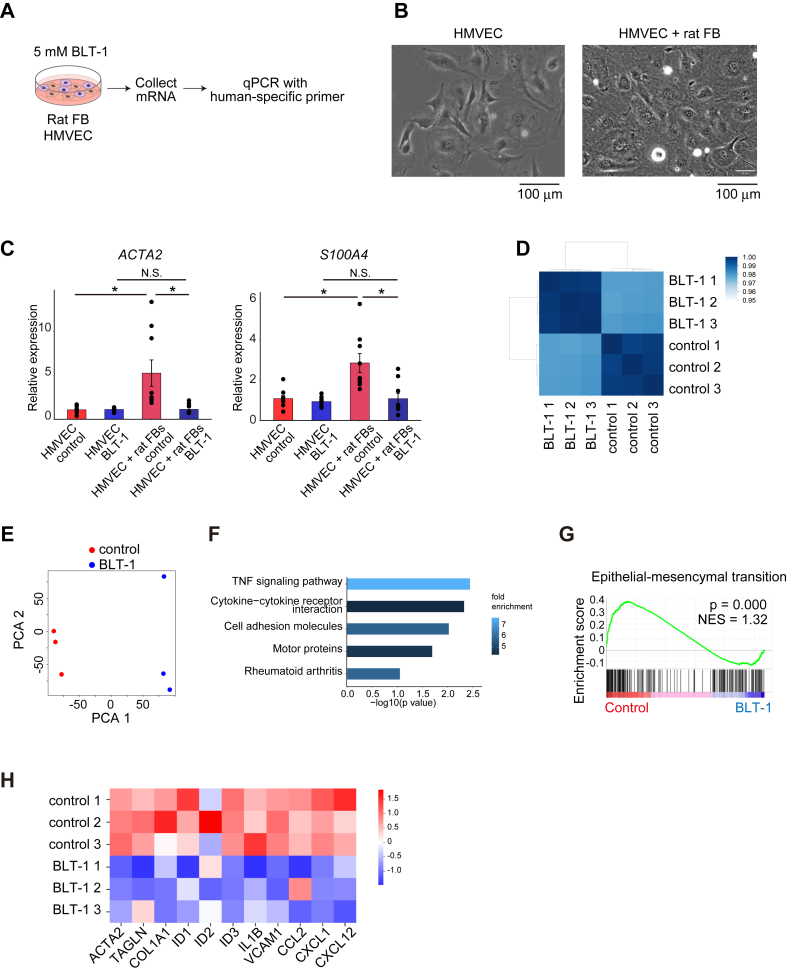
RNA-seq and Coculture Experiments Elucidated the Putative Cardioprotective Mechanisms of SCARB-1 Inhibition in ECs (A) Experimental schema of human EC and rat FB coculture system. (B) Human microvascular endothelial cells (HMVECs, left) and cocultured HMVECs/rat FBs (right). (C) Quantitative polymerase chain reaction (qPCR) analysis results of mixed coculture between HMVECs and rat FBs (n = 9). *GAPDH* was used as the internal control. (D) Correlation matrix heatmap of control/BLT-1-treated HMVEC RNA expression. Darker blue indicates a stronger correlation. (E) Principal component analysis (PCA) of RNA sequences for control and BLT-1 groups (n = 3). HMVECs were treated with dimethyl sulfoxide (control) or 1 mmol/L BLT-1 for 24 hours. (F) KEGG (Kyoto Encyclopedia of Genes and Genomes) pathway analysis result of down-regulated genes in BLT-1–treated group. (G) Characteristic results of gene set enrichment analysis of RNA sequences. The green line indicates the enrichment score. (H) Heatmap of representative differentially expressed genes between the control and BLT-1 treatment groups. Rows represent samples, and columns represent representative genes. Values were *z*-score. ∗*P* < 0.05. NES = normalized enrichment score; NS = not significant; TNF = tumor necrosis factor; other abbreviations as in Figures 1 and 5.

To confirm that FB-EC interaction mediated by SCARB1 caused pathologic changes in ECs, we performed mixed co-culture of FBs and ECs (Figures 6A and 6B). qPCR with human specific primers, which did not detect gene expressions of rat fibroblasts (Supplemental Figure 9A), revealed that mixed coculture of HMVECs and rat fibroblasts resulted in an increased expression of pathologic fibrotic markers such as *ACTA2* and *S100A4* in HMVECs, and treatment with BLT-1 decreased expression of these markers (Figure 6C). In contrast, in a separated coculture system, these gene expression changes were not observed (Supplemental Figure 9B), suggesting direct FB-EC crosstalk may be critical for pathologic changes in ECs. Next, to analyze expression changes in BLT-1–treated HMVECs, we performed RNA-seq analysis in these cells. Principal component analysis and clustering revealed distinct gene expression clusters in the control and BLT-1 groups (Figures 6D and 6E). KEGG pathway analysis indicated genes related to inflammatory pathway including tumor necrosis factor α signaling were enriched (Figure 6F). Gene set enrichment analysis showed a significant decrease in genes associated with epithelial-mesenchymal transition on BLT-1 administration (Figure 6G). Indeed, as shown in the heatmap, the expression of fibrotic markers (*ACTA2*, *TAGLN*, and *COL1A1),* proinflammatory molecules (*IL1B* and *VCAM1*), and chemokines (*CCL2*, *CXCL1*, and *CXCL12*) decreased following BLT-1 administration (Figure 6H). Genes related to BMP signaling (*ID1*, *ID2*, and *ID3*) were also decreased by BLT-1 (Figure 6H). We hypothesized THBS1 as potential ligands for SCARB1, as predicted based on interactome shown at Figures 3F and 3H. To validate the effect of THBS1 on the SCARB1 receptor, THBS1 was administered to HMVECs with or without BLT-1, followed by qPCR analysis. THBS1 treatment increased the expression of *IL1B* and *COL1A1*, and BLT-1 administration counteracted this increase (Supplemental Figure 9C). These genes were identified as important factors of pathologic ECs in scRNA-seq experiments using a TAC-induced heart failure mouse model (Figure 2C). These findings strengthen the results of in vivo analyses that pathologic changes during heart failure progression were caused by FB-EC interaction via SCARB1.

### Spatial omics analysis suggested underlying mechanism of heart failure progression through cell-cell interaction in the heart

Based on previous results, the importance of FB-EC interaction through SCARB1 receptor in heart failure progression was suggested. However, localization of cell-cell interaction in the actual heart and downstream mechanism of denatured ECs were not fully investigated.

**Figure 7:**
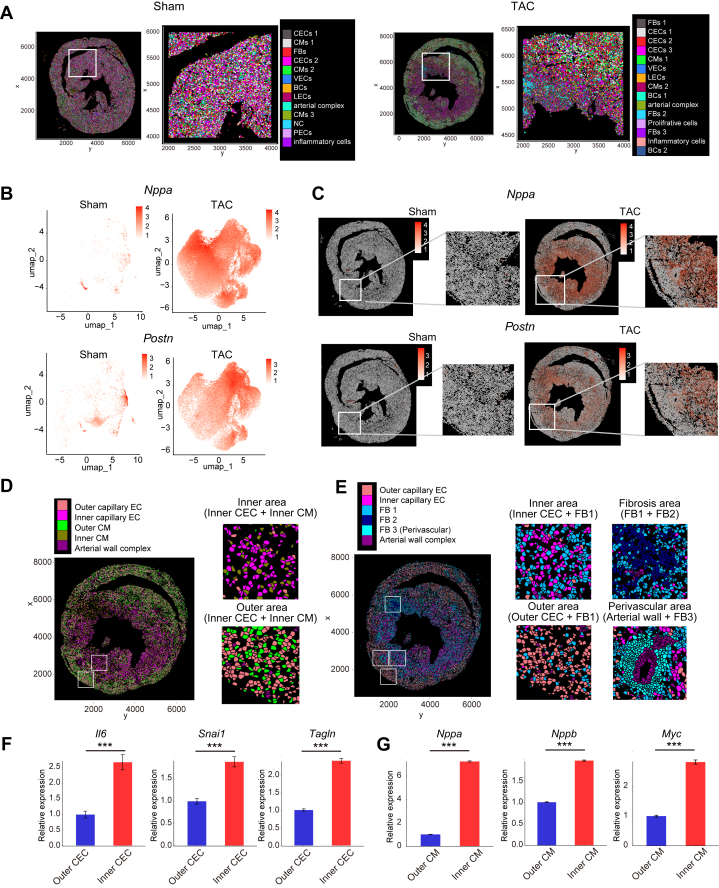
Spatial Omics Analysis Suggested Underlying Mechanism of Heart Failure Progression Through Cell-Cell Interaction in the Heart (A) Distribution of each cell type in the sham and TAC hearts. (B) Unique molecular identifier (UMI) counts visualization of *Nppa* and *Postn* in the sham and TAC groups. (C) UMI counts visualization projected on microscopic images. *Nppa* and *Postn* UMI count in the sham and TAC groups are shown. (D) Distribution of representative ECs and CMs in the TAC heart. (E) Distribution of representative ECs and FBs in the TAC heart. (F) Gene expression of *Il6*, *Snai1*, and *Tagln* in the outer CEC and inner CEC groups. Gene expression levels were normalized to outer CEC group. Wilcoxon rank-sum tests were conducted by “FindMarkers” function in Seurat package. (G) Gene expression of *Nppa*, *Nppb*, and *Myc* in the outer CM and inner CM groups. Gene expression levels were normalized to outer CM group. Statistical test was the same as (F). VEC, venous endothelial cell; other abbreviations as in Figures 1 and 2.

To clarify the spatial arrangement of cells in hearts including ECs and FBs, we conducted Xenium in situ spatial transcriptomic profiling of around 300 genes on hearts from sham and TAC 12 weeks (Figure 7A, Supplemental Figure 10A). The TAC heart exhibited higher expression of pathologic markers such as *Nppa* and *Postn* compared to sham (Figure 7B), primarily in the inner region (endocardial side) (Figure 7C), indicating a higher density of pathologic cells in the inner, relatively hypoxic area. Clustering cell populations by gene expression revealed uniform distribution in sham hearts (Figure 7A) but distinct inner vs outer regions (epicardial side) in TAC 12 weeks hearts (Figures 7A). Capillary endothelial cells (CECs) and CMs were notably segregated into inner and outer populations (Figure 7D). Inner CECs showed elevated inflammatory and fibrotic markers (*Il6*, *Snai1*, *Tagln*), consistent with our scRNA-seq (Figure 7F). Similarly, inner CMs had higher expression of heart failure–associated genes such as *Myc*, *Nppa*, and *Nppb* (Figure 7G). Pathological ECs and CMs were often in close proximity (Figure 7D), suggesting a functional relationship in heart failure progression. Whereas EC-CM interactions in diseased hearts have been previously reported,^24^^,^^25^ our data suggest that ECs influence CMs to induce heart failure. Focusing on EC and FB arrangement (Figure 7E), the inner region with abundant pathologic CECs also had higher FB1 population density, indicating that inner CEC-FB1 proximity is vital for interstitial fibrosis. In areas of severe fibrosis, FBs formed a distinct group (FB2) with reduced endothelial density, implying that vascular bed loss worsens fibrosis. Additionally, periarterial regions showed thickened arterial walls surrounded by another FB cluster (FB3) with minimal CM involvement. EC and FB distribution patterns suggest mutual influence between these cell types in the heart failure pathology. These findings enhance understanding of the underlying mechanisms of heart failure and may provide novel therapeutic strategies.

## Discussion

We clarified that FB-EC interactions through *Scarb1* are critical for EC pathologic changes during heart failure development, using scRNA-seq and spatial omics analysis. Supporting this, both SCARB1 inhibition and EC-specific *Scarb1* KO mitigated TAC-induced heart failure and cardiac fibrosis, identifying SCARB1 on ECs as a pivotal factor in heart failure progression and a potential therapeutic target. Despite BLT-1 administration and *Scarb1* CKO not preventing adaptive hypertrophy (Supplemental Figures 6C and 7B), they ultimately preserved cardiac function (Figures 4D and 5C), suggesting that blocking SCARB1 in ECs prevents the transition to heart failure. Moreover, mixed coculture system and THBS1 administration to HMVEC exhibited qualitative changes of ECs (Figures 6C to 6H, Supplemental Figures 9B and 9C), simulating an in vivo model. Thus, targeting the FB-EC interaction via SCARB1 could be promising for hypertensive heart failure treatment.

Whereas several studies have examined EC-CM or FB-CM interactions in healthy and diseased hearts,^18^^,^^20^^,^^22^ few prior investigations have explored the role of FB-EC interactions in the progression of heart failure. Our data emphasize the significance of intercellular interactions in the pathogenesis of hypertensive heart failure. scRNA-seq and intercellular interactome analysis suggested that FB-derived ligands bind to EC receptors, including SCARB1 (Figures 3E and 3F), thereby inducing pathologic changes characterized by inflammatory and fibrotic gene expression in ECs (Figure 2C). In mixed coculture experiments, key genes associated with these pathologic EC phenotypes were significantly up-regulated (Figure 6C), suggesting that FB-derived ligands promote EC pathogenesis. We further examined THBS1, one of the ligand candidates identified through interactome analysis, in EC monocultures and observed increased expression of inflammatory (*Il1b*) and fibrotic (*Col1a1*) genes, which was mitigated by BLT-1 treatment (Supplemental Figure 9C). These findings indicate that THBS1 is a crucial ligand for SCARB1, although additional ligands likely cooperate to drive EC pathology.

From the Xenium in situ results, FBs and ECs were spatially in close proximity in both sham and TAC hearts (Figures 7A). The TAC hearts showed higher expression of pathologic markers such as *Nppa* and *Postn*, primarily localized in the inner region (endocardial side) (Figures 7B and 7C). This area contained a higher density of pathologic ECs characterized by inflammatory and fibrotic gene expression, often in close proximity to CMs expressing *Myc*, *Nppa*, and *Nppb* (Figures 7D and 7G). Although the precise direction of these interactions remains unclear, these findings suggest that pathologically altered ECs may influence adjacent CMs and thereby exacerbate heart failure. The underlying factors driving the segregation of pathologic cells within the inner region were also not fully addressed in this study. However, within the TAC model, it is hypothesized that the inner myocardial region is subjected to hypoxic conditions,^54^ which may contribute to this cellular distribution. Additionally, regions exhibiting pronounced fibrosis demonstrated a significant reduction in EC density (Figure 7E). Similarly, in the hearts of TAC mice, vascular density was reduced compared to in control mice, and improvement was observed with *Scarb1* CKO (Figures 4I and 4J, Supplemental Figure 6E). These results indicate that microvascular rarefaction may worsen fibrotic remodeling or vice versa. Overall, our findings support a model where FB-derived ligands activate adjacent ECs, leading to inflammatory and fibrotic gene expression and endothelial dysfunction. Especially in the hypoxic inner region, these pathologic changes intensify in both ECs and CMs, potentially exacerbating heart failure. Additionally, advancing fibrosis deteriorates the vascular bed, further impairing blood flow and promoting adverse cardiac remodeling.

We also investigated the molecular mechanisms underlying these pathologic changes in ECs. Our scRNA-seq and spatial omics analyses revealed that inflammatory and fibrotic gene expression profiles are characteristic of ECs in heart failure (Figures 2C and 7F). Specifically, we observed that the tumor necrosis factor α and transforming growth factor β pathways might be activated downstream of the SCARB1 receptor (Figures 6F to 6H and 7F, Supplemental Figure 7C). The activation of these signaling pathways may mechanistically explain the exacerbation of heart failure. The role of endothelial-mesenchymal transition–like changes, induced by transforming growth factor β signaling, warrants particular attention (Figures 6G and 6H), as endothelial-mesenchymal transition is implicated in the progression of fibrosis following myocardial infarction^55^ and in various chronic diseases,^56^ including heart failure. Indeed, our data suggest that SCARB1 activation in ECs promotes an endothelial-mesenchymal transition–like state, and inhibition of SCARB1 reversed these phenotypes both in vitro and in vivo (Figures 4B to 4H, 5B to 5H, 6C, 6G, and 6H), highlighting the critical contribution of pathologic EC changes to heart failure progression. Furthermore, RNA-seq results indicate a potential involvement of BMP signaling (Figure 6H). Previous studies have demonstrated that BMP signaling is associated with atherosclerosis, calcification, and cardiac fibrosis.^56^^,^^57^ However, the current study does not provide sufficient data to conclusively support the role of BMP signaling in this context.

Our results appear to contrast with a prior study where systemic *Scarb1* deficiency exacerbated heart failure^58^; however, hepatocyte-specific *Scarb1* transfer yielded beneficial outcomes in the same study, underscoring the disparate functions of *Scarb1* in ECs and liver cells. Furthermore, we showed that specific inhibition of SCARB1 resulted in slight increase in HDL concentration. However, HDL uptake in the liver was not altered by SCARB1 inhibition (Supplemental Figures 8A to 8D), indicating that simple changes in HDL influx are insufficient to explain the mitigation of heart failure. Similarly, another study reported that EC-specific *Scarb1* KO mice exhibited reduced progression of atherosclerosis,^59^ which aligns with our cardioprotective findings. Consequently, we hypothesize that *Scarb1* expression in ECs, when stimulated by FBs, exacerbates heart failure due to enhanced fibrotic activity.

### Study limitations

The scRNA-seq experiments were conducted with only a single replicate for each phase. Nonetheless, we consider our findings reliable for several reasons. Changes in the known heart failure–specific FB populations^44^, ^45^, ^46^, ^47^, ^48^ (Figures 2E and 2G to 2H, Supplemental Figures 5A to 5B) provide partial validation of heart failure progression in our mouse model. Moreover, if adequate cell counts are obtained and sequencing quality is high, a single sample per stage can be deemed sufficient.^60^ Additionally, following the methodology of previous studies,^61^^,^^62^ we carefully validated the functions of SCARB1 in ECs in vivo and in vitro to support the scRNA-seq data. Finally, we complemented these data with spatial transcriptomic analyses and in vivo/in vitro functional assays, thereby solidifying the link between SCARB1 in ECs and heart failure progression. However, the precise mechanisms and ligands of SCARB1 in interactions between ECs and FBs remain elusive. Additional research is required to elucidate the roles of SCARB1 in pathologic endothelial changes and to develop targeted cardiovascular therapies. Despite these limitations, we believe that our findings contribute significantly to understanding the role of ECs in heart failure progression and to the development of innovative cardiovascular therapies. Continued research based on these insights may provide significant benefits for future clinical applications.

Perspectives **COMPETENCY IN MEDICAL KNOWLEDGE:** According to current clinical guidelines, there are limited pharmacologic interventions available to prevent exacerbation of hypertensive cardiomyopathy, which is the major etiology of heart failure. This study utilizes scRNA-seq and machine learning technologies to demonstrate that interactions between ECs and FBs mediated by SCARB1 receptors on ECs represent a viable target for managing progressive heart failure with cardiac hypertrophy. The SCARB1-specific inhibitor BLT-1 has been shown to exert a cardioprotective effect in this context.

**TRANSLATIONAL OUTLOOK:** BLT-1, which targets SCARB1 on endothelial cells, shows promise as a novel therapeutic strategy for patients with hypertensive heart failure. Further investigations into the mechanisms of action, efficacy, and safety of BLT-1 administration are essential to develop this compound into a practical medication for heart failure treatment.

## Funding Support and Author Disclosures

This research was supported by AMED under grant 22ek0210169h0001, Bayer Academic Support, MSD Life Science Foundation, Japanese Heart Failure Society, and Takeda Science Foundation. Dr Fukuda has reported that he is a Founding Scientist funded by the SAB of Heartseed Co, Ltd. All other authors have reported that they have no relationships relevant to the contents of this paper to disclose.
