# Supplementary material for: Inhibition of Scarb1 on Endothelial Cells Attenuates Pressure Overload-Induced Heart Failure Progression
Source: JACC Basic Transl Sci. 2025 Aug 6;10(10):101308. doi: 10.1016/j.jacbts.2025.05.003 (PMC12665495; doi:10.1016/j.jacbts.2025.05.003)
Supplement: Supplemental Figures 1-10 [file mmc1.docx]

**Supplemental information**

**Supplemental figures**

**Supplemental Figure 1. Acquiring single-cell expression profile of failing hearts**

**(A)** Murine heart cells were dissociated by collagenase and separated by Percoll. The yellow arrow indicates the boundary where non-cardiomyocytes were concentrated. **(B)** Microscopic photograph of the previous cells. The cells were then resuspended in PBS. **(C)** Summary of scRNA-seq results for healthy, adaptive hypertrophic, and failed heart samples. **(D)** Expression levels of housekeeping genes displayed in violin plots. *Actb* (left) and *Gapdh* (right). **(E)** Right and left carotid artery doppler velocity of representative Sham and TAC mice **(F)** PWT, IVS and HW/BW parameters of representative Sham and TAC mice. HW/BW was tested using Wilcoxon rank-sum test. **(G)** Heatmap of characteristic gene expression in each cell type. Rows indicate genes. Columns represent cell types. Brighter colors indicate stronger expression. **(H and I)** Cell numbers annotated in these experiments. Rows indicate cell types. The columns represent the disease progression phases. **(H)** Crude cell number and **(I)** proportion. PWT, posterior left ventricular wall thickness; IVS, interventricular septum thickness; HW/BW, heart weight divided by body weight; PBS, phosphate-buffered saline.

**Supplemental Figure 2. Detailed subtypes of endothelial cells**

**(A and B)** Annotated cell numbers in EC subgroups through heart failure progression. Rows indicate cell types. The columns represent the disease progression phases. **(A)** Crude cell numbers and **(B)** proportions. **(C)** Proportional fold-changes of each EC subgroup cell number in adaptive hypertrophy and heart failure compared to the healthy state.

EC, endothelial cells.

**Supplemental Figure 3. Characterization of endothelial cells during heart failure progression**

**(A)** GO analysis graphs (left: cellular component; right: molecular function) in EC subgroup 6. The rows in the GO graphs were top-10 significant GO terms. **(B)** UMAP in EC subgroup 5. In the UMAP plot, pink, green, and blue dots indicate healthy, adaptive hypertrophy, and failed heart cells, respectively. **(C)** GO analysis graph (left: cellular component; right: KEGG pathway graph) of the heart failure phase compared to the healthy heart in EC subgroup 5. EC, endothelial cell; GO, gene ontology analysis; KEGG, Kyoto Encyclopedia of Genes and Genomes.

**Supplemental Figure 4. Delineating fibroblast characteristics at the single-cell level**

**(A and B)** Number of annotated cells in FB subgroups through heart failure progression. Rows indicate cell types. The columns represent the disease progression phases. **(A)** Crude cell numbers and **(B)** proportions. **(C)** Proportional fold-changes of each FB subgroup cell number in adaptive hypertrophy and heart failure compared to the healthy state. FB, fibroblasts.

**Supplemental Figure 5. Characterization of fibroblasts during heart failure progression**

**(A and B)** Visualization of GO analysis (left: biological process; right: KEGG pathway) in **(A)** activated fibroblasts and **(B)** myofibroblasts. Rows are top-5 significant GO terms. GO, gene ontology analysis; KEGG, Kyoto Encyclopedia of Genes and Genomes; FDR, false discovery rate.

**Supplemental Figure 6. Differences between control and *Scarb1* CKO TAC mice**

**(A)** *Scarb1* RNA expression profiles of control and *Scarb1* CKO mice (*n* = 3). Y-axes are relative expression levels to internal *Gapdh*. Blue/red bars represent control/ *Scarb1* CKO TAC mice. **(B)** Representative microscopic images of HE-stained control/*Scarb1* CKO TAC mouse specimens. Control (left) and *Scarb1* CKO (right). **(C)** Temporal trends in echocardiographic parameters in control/*Scarb1* CKO TAC mice. Blue lines represent control mice graphs, and red lines represent *Scarb1* CKO. The x-axis represents weeks after TAC. LVDs (leftmost), LVDd (second left), IVS (second right), and PWT (rightmost). The unit of the y-axis is millimeters. **(D)** LVEF in the TAC control and TAC *Scarb1* CKO groups at week 0 and week 12. Wilcoxon rank-sum test was used for the data at week 12. **(E)** Vascular count density of the Sham, TAC control, and TAC *Scarb1* CKO groups. **(F)** HW/BW in the TAC control and TAC *Scarb1* CKO groups at week 12. **(G)** Survival curves of TAC control and TAC *Scarb1* CKO mice. The dots represent raw data. CKO, conditional knockout; TAC, transverse aortic constriction; LVEF, left ventricular ejection fraction; HW/BW, heart weight divided by body weight.

**Supplemental Figure 7. Differences between control and BLT-1-treated TAC mice**

**(A)** Representative microscopic images of HE-stained control/BLT-1-treated TAC mice. Control (left) and BLT-1-treated (right). **(B)** Temporal trends in echocardiographic parameters in control/BLT-1-treated TAC mice. Blue lines represent control mice graphs, and red lines represent BLT-1-treated mice. The x-axis represents weeks after TAC. The LVDd (leftmost), LVDs (second left), IVS (second right), and PWT (rightmost). The unit of the y-axis is millimeters. **(C)** RNA expression profiles of TAC control, and BLT-1-treated TAC mice (*n* = 9 and 6, respectively). Y-axes are expressed relative to internal *Gapdh*. blue/red bars represent TAC control/BLT-1-treated TAC mice. **(D)** HW/BW in the TAC control and TAC *Scarb1* CKO groups at week 8. The **(E)** Survival curves of TAC control and TAC BLT-1 treated mice. The dots represent raw data. BLT-1, block lipid transport-1; TAC, transverse aortic constriction; HE, hematoxylin and eosin; LVDd, left ventricular end-diastolic diameter; LVDs, left ventricular end-systolic diameter; IVS, interventricular septum thickness; PWT, posterior wall thickness.

**Supplemental Figure 8. HDL dynamics and BLT-1 effect in vivo**

**(A)** Experimental schematic of Dil-HDL injection to visualize in vivo HDL dynamics. Dil-HDL 30 µg/body was retro-orbitally injected to sample mice, and they were sacrificed after 6 h. **(B)** Serum HDL concentration in control and BLT-1 mice (*n* = 3). PBS (1 mL) was intraperitoneally injected into the control mice for 7 days before sacrifice. BLT-1 (1.25 mg DMSO/PBS) was injected into the treatment group of mice in the same manner. Wilcoxon rank-sum test was used to test these data. **(C and D)** Immunohistochemistry (IHC) images of control/BLT-1-treated mice and Dil-HDL-positive cell counts. Liver control IHC (C, left), BLT-1-treated IHC (C, middle), and Dil-HDL-positive cell counts (C, right). Control heart IHC (D, left), BLT-1-treated IHC (D, middle), and Dil-HDL-positive cell counts (D, right). No Dil-HDL-positive cells were detected in heart samples. HDL, high-density lipoprotein; BLT-1, block lipid transport-1; Dil-HDL, 1,1'-3,3,3',3'-tetramethylindocarbocyanine perchlorate-labeled HDL; PBS, phosphate-buffered saline.

**Supplemental Figure 9. indirect co-culture between HMVEC and rat fibroblasts**

**(A)** qPCR analysis results of HMVEC and rat fibroblasts using human specific primers (*n* = 9). **(B)** qPCR analysis results of monoculture HMVEC and separate co-culture HMVEC using human specific primers (*n* = 9). **(C)** qPCR analysis results of *IL1B* (*n* = 3) and *COL1A1* (*n* = 6) in control HMVEC, THBS1 administered HMVEC without BLT-1, and THBS1 administered HMVEC with BLT-1 groups. Y-axes are expressed relative to internal *GAPDH*. The dots represent raw data. Wilcoxon rank-sum test was used to test these data. qPCR, quantitative polymerase chain reaction; HMVEC, human cardiac microvascular endothelial cells; THBS1, thrombospondin-1.

**Supplemental Figure 10. Spatial omics analysis of Sham and TAC hearts**

**(A)** Microscopic image of Sham heart (left) and TAC heart (right), used for spatial omics analysis, and UMAP plot for segmented cells of spatial omics analysis.
